# Inappropriate placement of urinary catheters into the ureter: A case report and literature review

**DOI:** 10.1097/MD.0000000000037623

**Published:** 2024-04-12

**Authors:** Jiun-Jia Li, Chin-Fong Au

**Affiliations:** a Division of Urology, Department of Surgery, Far Eastern Memorial Hospital, New Taipei City, Taiwan

**Keywords:** Neurogenic bladder, suprapubic catheter, ureteral cannulation, ureteral misplacement, urosepsis

## Abstract

**Objective:**

To emphasize preventive measures and outline management strategies for inadvertent ureteral cannulation.

**Methods:**

We present a case report and conduct a literature review of 39 case reports on ureteral cannulation, examining a total of 48 patients.

**Results:**

About 67% of the cases were female, and long-term catheterization was observed in 67% of the cases. Neurological conditions such as spinal cord injury (SCI), stroke, dementia, multiple sclerosis, and myasthenia gravis were the primary factors (48%) in ureteral cannulation. Symptoms included flank pain (46%), fever (31%), oliguria (27%), non-deflatable balloon issues (25%), hematuria (21%), abdominal pain (17%), urine leak (12.5%), and nausea/vomiting (8%). Complications varied, including acute pyelonephritis (35%), acute kidney injury (27%), urosepsis (21%), and ureter rupture (17%). Despite inadvertent catheter placement, 25% of patients had no complications. More than half of the patients (58%) were managed through catheter change, while 27% underwent cysto-ureteroscopy with or without balloon puncture or ureteral stenting. Additionally, 10% received interventional radiology procedures, 6.25% underwent surgical repair, and 4% underwent ultrasound-guided balloon puncture.

**Conclusions:**

Female gender, neurologic conditions, and long-term catheterization were identified as predominant risk factors. Early detection of ureteral cannulation can prevent severe complications, particularly in certain special populations such as patients with neurogenic bladder or SCI, who may have reduced sensation and expression capabilities.

## 1. Introduction

Urinary catheterization is clinically widely performed and is considered a comparatively safe procedure. However, the misplacement of a catheter into the ureter, which leads to serious complications, is considered troublesome. Herein, we present a case report and conduct a literature review of 39 case reports on ureteral cannulation, examining a total of 48 patients.

## 2. Case report

### 2.1. Patient Information

A 82-year-old man with a history of prostate cancer and stroke, who is completely dependent on activities of daily living (ADLs), visited our emergency department due to shortness of breath and a spiking fever for the past 2 days. He had a suprapubic catheter (SPC) created 8 years ago for the neurogenic bladder. A routine SPC change (16 Fr. Foley Catheter) by a urologist 1 week before he visits the emergency department. Upon arrival, his vital signs were as follows: body temperature of 39.1°C, heart rate of 132/min, blood pressure of 82/52 mm Hg, respiratory rate of 26/min, with oxygen saturation of 97% on a 6 L/min O_2_ mask. A preliminary diagnosis of septic shock was made. His physical examination findings were unremarkable, and he was unable to express himself well due to history of stroke and current bedridden status. Low urine output was discovered by his family after the SPC was changed.

### 2.2. Diagnostic finding

**Figure 1.**
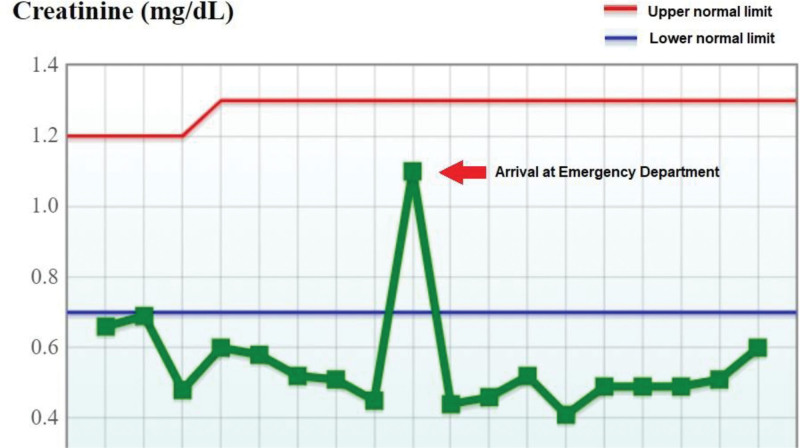
The patient’s serum creatinine levels over time.

**Figure 2.**
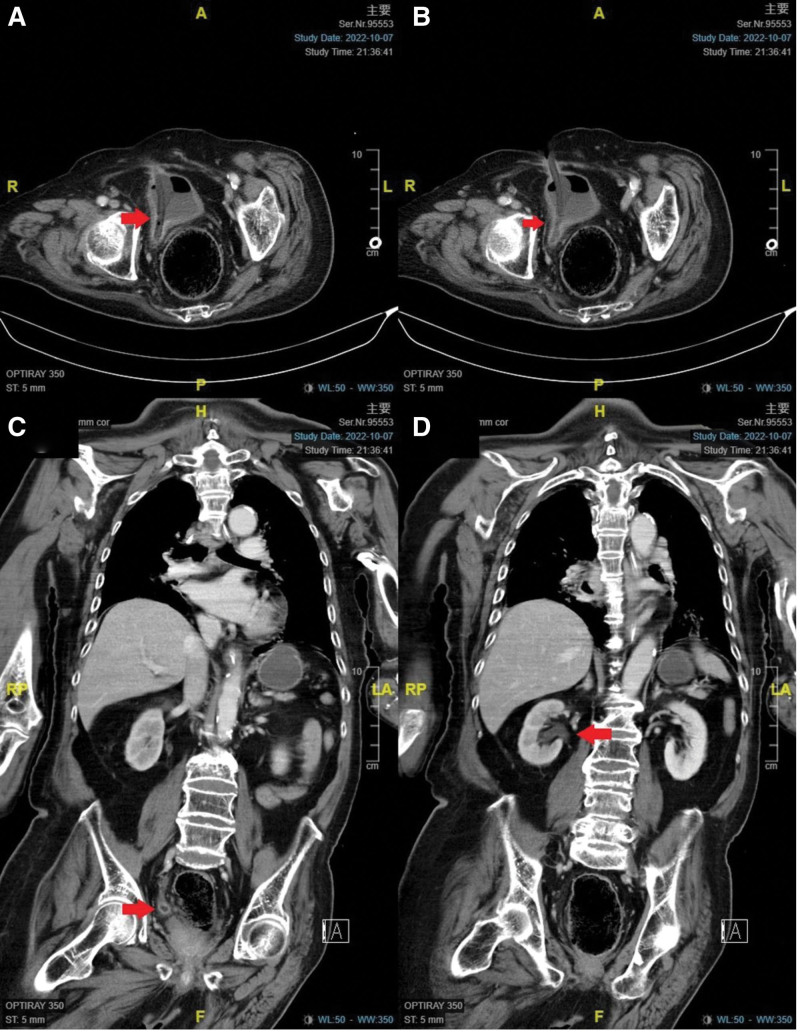
Abdomen and pelvic CT revealed (A–C) misplacement of the SPC tip (arrow) into the right ureter (D) right hydronephrosis (arrow) with perinephric fat stranding.

Blood test revealed elevated white blood cells (WBC): 19,730/μL with a left shift, lactate: 5.02 mmol/L, C-reactive protein: 6.18 mg/dL, and Na: 122 mmol/L. His renal function was impaired, with a creatinine of 1.10 mg/dL compared to his baseline of 0.5 mg/dL (Fig. 1). Urinalysis showed red blood cells: 276/HPF, WBC: 203/HPF, and bacteria: 75.83 * 10^5^/mL. Abdominal and pelvic computed tomography (CT) was performed, revealing pneumonia and misplacement of the SPC tip into the right ureter, causing renal pelvis dilatation and perinephric fat stranding (Fig. 2).

### 2.3. Management

The SPC was deflated and replaced with another 16 Fr. catheter, immediate ultrasound was conducted to confirm the balloon position. The patient required inotropic support for an additional 36 hours, and an empirical antibiotic (piperacillin-tazobactam) was administered for pneumonia and pyelonephritis. His urine culture revealed *Enterococcus faecalis* and *Acinetobacter baumannii* complex (XDRAB). Piperacillin-tazobactam was replaced by colistin on day 10. He was discharged after a 25-day hospital course.

## 3. Literature Review

Urinary catheterization is clinically widely performed and is considered a comparatively safe procedure, particularly among patients with neurogenic bladder. Catheter-related minor complications, such as urinary tract infections (UTIs), bleeding, urethral injury, catheter malfunction, entry into a false route, and retention of the catheter balloon in the urethra, are reported.^[1,2]^ Serious complications rarely occur, and the misplacement of a urinary catheter into the ureter, leading to ureter rupture is considered troublesome. Iatrogenic or externally induced trauma are the predominant causes of ureteral injuries, with the former arising from urologic procedures (42%), gynecologic interventions (34%), and general surgical interventions (24%).^[3,4]^ Inflation of a misplaced catheter balloon within the ureter could cause ureter rupture, which can lead to numerous complications, including abscess formation, urinomas, and urosepsis, requiring prompt evaluation and intervention.^[5]^

This was the only case of a SPC misplaced into the ureter from our hospital, impelling us to review the literature for similar cases. We searched the published case reports and literature reviews, encompassing 39 case reports on ureteral cannulation. A total of 48 patients were examined, and our reviews are the most integrated to date. The details of these cases are listed in Table 1.^[6–44]^ Patient demographics and clinical characteristics are summarized in Tables 2 and 3.

**Table 1:** Detailed information of the cases included in the literature review.

Author	Year	Case	Age (yr)	Sex	UC/SPC	Side	Underlying disease	Long-term catheter	Fr.	ADLs	NB/CB	Presentation	Diagnosis	Ureter rupture	Consequences				Management
															APN	Urosepsis	AKI	Others	
Singh and Eardley^[6]^	1996	1	26	F	UC	R	Spina-Bifida (Paraplegia)	Y	14	Y	Y	Incidental	Intraoperative (ileal conduit)	+					Surgical repair
Kato^[7]^	1997	1	74	F	UC	L	Rheumatoid arthritis, VUR	Y	16	Y	Y	Non-deflatable balloon	Fluoroscopy						Catheter change
Muneer et al^[8]^	2002	1	77	M	UC	R	Bladder cancer, CKD, VUR	Y	–	N	Y	Peri-catheter urine leak, flank pain, non-deflatable balloon	Cysto-ureteroscopy				+		Cysto-ureteroscopy, balloon puncture
Hara et al^[9]^	2005	1	51	F	UC	L	SUI	N	16	N	N	Peri-catheter urine leak, flank pain	Fluoroscopy						Cysto-ureteroscopy, DJ, catheter change
George and Tharion^[10]^	2005	1	14	M	UC	R	Acute demyelinating encephalomyelitis (ADEM) with T6 paraplegia	Y	16	Y	Y	Peri-catheter urine leak, flank pain	US						Catheter change
Wang and Foote^[11]^	2006	1	30	F	UC	R	Pregnant (vaginal delivery)	N	14	N	N	Peri-catheter urine leak, flank pain, non-deflatable balloon	Cysto-ureteroscopy						Cysto-ureteroscopy, balloon puncture
Maegele et al^[12]^	2006	1	86	F	UC	L	N/A	N/A	–	N/A	N/A	Flank pain, fever, vomiting	CT		+				Catheter change
Kim and Park^[13]^	2008	1	38	F	UC	R	SCI (T10 Paraplegia)	Y	14	Y	Y	Low abdominal pain	Fluoroscopy	+					Nephrostomy
Papacharalabous et al^[14]^	2009	1	68	F	UC	L	Gynecologic cancer	N	14	N	Y	Incidental	Intraoperative (debulking surgery)	+					Surgical repair
Dangle et al^[15]^	2010	1	50	F	SPC	L	Multiple sclerosis, solitary kidney	Y	–	Y	Y	Flank pain, oliguria	CT				+		Catheter change
Hale et al^[16]^	2012	1	80	F	UC	L	N/A	N	16	N	N	Flank pain, vomiting	CT	+					Surgical repair
Adeyemo et al^[17]^	2013	1	55	M	SPC	R	SCI (C6 Quadriplegia), Urethral stricture	Y	30	Y	Y	Altered mental status	CT			+			Catheter change
Baker et al^[18]^	2013	1	59	F	UC	L	Multiple sclerosis	Y	16	Y	Y	Oliguria, vomiting, hematuria	CT	+	+			Mortality, unrelated sepsis (osteomyelitis)	Interventional Radiology, Antegrade DJ
Anderson and Greenlund^[19]^	2013	1	48	F	UC	R	SCI (C6 Quadriplegia)	Y	-	Y	Y	Flank pain, hematuria	CT					Hypertension Crisis (206/129 mm Hg)	Catheter change
		1	68	M	UC	R	SCI (C4-5 Quadriplegia)	Y	–	Y	Y	Low abdominal pain, hematuria	CT						Catheter change
Modi et al^[20]^	2014	1	83	F	UC	R	Multiple sclerosis	Y	–	Y	Y	Flank pain	N/A	+					Interventional Radiology, Antegrade DJ, Nephrostomy
Ishikawa et al^[21]^	2014	1	81	M	UC	L	Atrophic bladder	Y	–	N/A	Y	Fever	CT		+				Catheter change
		1	67	F	UC	L	SLE, Stroke	Y	–	N/A	Y	Fever	CT		+				Catheter change
		1	37	F	UC	L	Paraplegia	Y	16	Y	Y	Peri-catheter urine leak, flank pain	CT						Catheter change
Viswanatha et al^[22]^	2014	1	N/A	F	UC	R	Pregnant (C/S)	N	–	N	N	Flank pain, non-deflatable balloon	Fluoroscopy						Cysto-ureteroscopy, Balloon Puncture
Crawford et al^[23]^	2015	1	86	F	UC	R	Interstitial cystitis	Y	–	N	Y	Flank pain, oliguria	CT		+	+	+		Cysto-ureteroscopy, DJ
Choi et al^[24]^	2016	1	63	M	SPC	R	SCI (C7 Quadriplegia)	Y	–	Y	Y	Fever, hematuria	CT	+	+				Nephrostomy
McGregor et al^[25]^	2016	1	28	F	UC	R	Pregnant (vaginal delivery)	N	14	N	N	Oliguria, non-deflatable balloon	Cysto-ureteroscopy						Cysto-ureteroscopy, Balloon Puncture, DJ
Onuigbo et al^[26]^	2017	1	41	F	UC	R	Multiple sclerosis	Y	–	Y	Y	Flank pain, fever, non-deflatable balloon	CT, cysto-ureteroscopy		+		+		Cysto-ureteroscopy, Balloon Puncture
Luo et al^[27]^	2017	1	85	M	UC	R	L2-3 Cystic Schwannoma	Y	–	N/A	Y	Flank pain, fever	CT		+	+			Catheter change
		1	74	F	UC	R	Gynecologic cancer, R/T	Y	–	N	Y	Oliguria	CT					Unrelated sepsis (Pneumonia)	Cysto-ureteroscopy, catheter change
		1	47	F	UC	L	AUR, Schizophrenia, antipsychotic medication	Y	–	N	N	Oliguria	CT				+		Catheter change
Agarwal et al^[28]^	2018	1	30	F	UC	L	SCI (T8 Paraplegia)	Y	16	Y	Y	Fever, oliguria, non-deflatable balloon, hematuria	CT		+		+		Ultrasound-guided balloon puncture
Shuaibin et al^[29]^	2018	1	80	M	SPC	L	Stroke, Senile dementia, TURP hx	Y	–	N	N	Fever, flank pain, hematuria	CT		+				Catheter change
Ogawa et al^[30]^	2018	1	86	F	UC	R	Lumbar compression fracture, osteoporosis	Y	–	Y	Y	Low abdominal pain, hematuria, peri-catheter urine leak	CT		+				Cysto-ureteroscopy
Löcherbach et al^[31]^	2018	1	64	F	UC	L	Left renal stone	N	12	N	N	Incidental	Intraoperative (cysto-ureteroscopy)	+					Cysto-ureteroscopy, DJ
Smekal et al^[32]^	2020	1	18	F	UC	R	Pregnant (vaginal delivery)	N	12	N	N	Flank pain, non-deflatable balloon	CT					Urinary diversion with SPC	Cysto-ureteroscopy, balloon puncture
Hijazo-Gascón et al^[33]^	2020	1	85	M	UC	R	Myasthenia Gravis	Y	–	N	N	Low abdominal pain, oliguria	CT		+	+	+		Catheter change
		1	75	M	UC	R	Bladder cancer S/P TUR Resection, VUR	Y	–	N	Y	Low abdominal pain, hematuria	CT						Catheter change
Cho et al^[34]^	2021	1	43	M	UC	R	Lung cancer	N	16	N	N	Flank pain, oliguria, general weakness, non-deflatable balloon	CT					Mortality due to cancer progression	Catheter change
Saad and Zhong ^[35]^	2021	1	73	M	SPC	R	SCI (C6-7 Quadriplegia)	Y	–	Y	Y	Low abdominal pain, Hematuria	CT						Catheter change
Chakra et al^[36]^	2022	1	58	F	UC	L	Neurogenic bladder	N/A	–	N	Y	Fever	N/A			+			Catheter change
Danaie et al^[37]^	2022	1	19	F	UC	R	Pregnant (vaginal delivery)	N	16	N	N	Flank pain, oliguria	CT				+		Catheter change
Ngoo and Hirst^[38]^	2022	1	61	M	SPC	R	Urethral stricture	Y	16	N	N	Flank pain, fever	CT			+	+		Catheter change
Al-Zubi et al^[39]^	2022	1	76	F	UC	R	Benign brain tumor, diabetes	Y	–	N	Y	Flank pain, oliguria	CT						Catheter change
Calderon Plazarte et al^[40]^	2022	1	66	M	SPC	L	Urethral stricture, TURP hx	Y	12	N	N	Flank pain, fever, oliguria	CT		+	+	+		Catheter change
		1	59	F	UC	L	Rectal cancer S/P LAR, CCRT	Y	16	N	N	Low abdominal pain, hematuria, nausea	CT		+		+		Catheter change
Qin et al^[41]^	2022	1	86	M	UC	R	Bladder cancer S/P TUR Resection, Bladder instillation	N	18	N	Y	Low abdominal pain, non-deflatable balloon	CT						Ultrasound-guided balloon puncture
Sturgess and Gill^42]^	2023	1	85	M	UC	L	Bladder cancer S/P TUR Resection, Bladder instillation	N	–	N	Y	Non-deflatable balloon	CT						Cysto-ureteroscopy, balloon puncture
Ito et al^[43]^	2023	1	74	F	UC	R	Polyarteritis Nodosa	Y	–	N	Y	Fever	CT		+	+			Catheter change
Patel et al^[44]^	2023	1	11	F	UC	L	N/A	N	–	N	N	Oliguria, fever	US		+				Catheter change
		1	7	F	UC	R	Cleft palate, Patent ductus arteriosus	N	–	N	N	Fever, flank pain, non-deflatable balloon	US			+	+		Cysto-ureteroscopy, balloon puncture, Nephrostomy
Our case	2023	1	82	M	SPC	R	Stroke, prostate cancer, urethral stricture	Y	16	Y	Y	Fever	CT		+	+	+		Catheter change

ADLs = Activities of daily living Dependent, AKI = acute kidney injury, APN = acute pyelonephritis, CKD = chronic kidney disease, CT = computed tomography, DJ = double-J stent, F = female, Fr. = French, L = left, M = male, NB/CB = neurogenic/contracted bladder, R = right, SCI = spinal cord injury, SPC = suprapubic catheter, UC = urethral catheter, US = ultrasonography, VUR = vesicoureteral reflux.

**Table 2:** Patient demographics of ureteral cannulation in the literature review (39 case reports).

		Patients (*N* = 48)
Median age (Range), y		64 (7–86)
Gender- no. (%)		
	Male	17 (35%)
	Female	31 (65%)
Catheterization- no. (%)		
	Urethral	40 (83%)
	Suprapubic	8 (17%)
Side- no. (%)		
	Left	19 (40%)
	Right	29 (60%)
Long-term catheter- no. (%)		
	Yes	32 (67%)
	No	14 (29%)
Catheter size (French Fr.)- no. (%)		
	Fr.12	3 (6%)
	Fr.14	5 (10%)
	Fr.16	12 (25%)
	Fr.18	1 (2%)
	Fr.30	1 (2%)
	N/A	23 (48%)
Underline disease- no. (%)		
	Spinal cord injury (Paraplegia or Quadriplegia), Lumbar Cystic Schwannoma	11 (23%)
	Neurological disorder (Stroke, Dementia, Spine compression fx, psychological hx, MG)	8 (17%)
	Pregnancy	5 (10%)
	Multiple sclerosis	4 (8%)
	Pelvic cancer ± R/T	4 (8%)
	Urethral stricture	4 (8%)
	Bladder cancer (TUR Resection, Bladder Instillation)	4 (8%)
	Vesicoureteral reflux	4 (8%)
	Immune disease (SLE, Rheumatoid arthritis, Polyarthritis Nodosa)	3 (6%)
Activity of daily living (ADLs)- no. (%)		
	Dependent	18 (38%)
	Independent	27 (56%)
Neurogenic/contracted bladder- no. (%)		
	Yes	30 (63%)
	No	16 (33%)

**Table 3:** Clinical characteristics of ureteral cannulation in the literature review (39 case reports).

	Patients (*N* = 48)
Clinical presentations- no. (%)	
Flank pain	22 (46%)
Fever	15 (31%)
Oliguria	13 (27%)
Non-deflatable balloon	12 (25%)
Hematuria	10 (21%)
Low abdominal pain	8 (17%)
Peri-catheter urine leak	6 (12.5%)
Nausea/vomiting	4 (8%)
Incidental (intraoperative)	3 (6.25%)
Altered mental status	1 (2%)
Diagnostic tools- no. (%)	
Computed tomography (CT)	33 (69%)
Cysto-ureteroscopy	5 (10%)
Fluoroscopy	4 (8%)
Incidental (intraoperative)	3 (6.25%)
Ultrasonography	3 (6.25%)
Consequences- no. (%)	
Acute pyelonephritis	17 (35%)
Acute kidney injury (AKI)	13 (27%)
Uneventful	12 (25%)
Urosepsis	10 (21%)
Ureter rupture	8 (17%)
Hypertension crisis	1 (2%)
Mortality	0 (0%)
Management- no. (%)	
Catheter change	28 (58%)
Cysto-ureteroscopy ± balloon puncture	13 (27%)
Interventional radiology (Antegrade DBJ or Nephrostomy)	5 (10%)
Surgical repair	3 (6.25%)
Ultrasound-guided balloon puncture	2 (4%)

## 4. Results

The median age was 64 years (range, 7–86 years). About 67% of the cases were female, and it is noteworthy that there were 3 pediatric cases. Regarding the side of cannulation, 40% were on the left side, and 60% on the right. Long-term catheterization was observed in 67% of the cases, with 29% not requiring a long-term catheter. The catheter sizes, with the majority (48%), are not specified (N/A). Among specified sizes, 16 Fr. catheters were most common (25%), followed by 14 Fr. (10%) and 12 Fr. (6%).

Neurologic conditions (spinal cord injury [SCI], stroke, dementia, multiple sclerosis, myasthenia gravis) played a predominant role (48%) in ureteral cannulation, emerging as the most significant factor among the underlying conditions examined. They contributed nearly half (23/48) of the cases, followed by pregnancy (10%). Other risk factors are detailed in Table 2. Additionally, 63% of patients had neurogenic or contracted bladders, while 33% did not. In terms of the patients’ ADLs, 38% were dependent, and 56% were independent. It’s important to note that ADLs may not directly correlate with ureteral cannulation.

Nearly half of the patients (46%) experienced flank pain, while 31% presented with fever. Other clinical manifestations included oliguria (27%), non-deflatable balloon issues (25%), hematuria (21%), low abdominal pain (17%), peri-catheter urine leak (12.5%), and nausea/vomiting (8%).

CT is the most commonly used diagnostic tool for evaluation (69%), followed by cysto-ureteroscopy (10%), and fluoroscopy (8%).

Consequences of ureteral cannulation were diverse, with acute pyelonephritis affecting 1-third of patients (35%), acute kidney injury (AKI) in 27%, urosepsis in 21%, ureter rupture in 17%, and no related mortality. The only 2 mortalities were due to unrelated sepsis (lung cancer progression and osteomyelitis). Despite the inadvertent placement of urinary catheters, 1-quarter of patients (25%) underwent the procedure without any complications or adverse events.

More than half of the patients (58%) were managed through catheter change, while 27% underwent cysto-ureteroscopy with or without balloon puncture or ureteral stenting. Additionally, 10% received interventional radiology procedures (antegrade double-J stent placement or nephrostomy), 6.25% underwent surgical repair, and 4% underwent ultrasound-guided balloon puncture. Two out of 3 cases with ruptured ureters, discovered incidentally during the operation, underwent surgical repair. One case received debulking surgery, while the other underwent ileal conduit surgery.

## 5. Discussion

Risk factors for unintended ureteral cannulation have been delineated in the literature^[27,31]^:

1. *Female anatomy.* The female urethra, being shorter than the male urethra, may be susceptible to misdirection during catheter insertion due to the angle at which the catheter enters the bladder. A similar theory applies to SPC.
2. *Pregnancy-related changes.* Physiological alterations during pregnancy, attributed to elevated progesterone levels, are recognized for inducing the dilatation of the ureter.^[45]^
3. *Neurological disorder (SCI, stroke, dementia, multiple sclerosis):* SCI can result in an upper motor neuron syndrome, leading to bladder hyperactivity characterized by spasms, urgency, and incontinence. Over time, this can lead to reduced bladder capacity and heightened bladder pressures, contributing to increased vesicoureteral reflux. The delayed presentation is attributed to the sensory dysfunction of the bladder,^[46,47]^ patients’ inability to feel pain or communicate, resulting in acute pyelonephritis (30%) and AKI (28%).
4. *Long-term catheter:* The bladder tends to contract and decrease its capacity, and frequent catheter changes, as a result, they have more opportunities for the catheter to be misplaced into the ureter.^[27]^
5. *Neurogenic/contracted bladder:* Vesicoureteral reflux with patulous ureteral orifices may facilitate catheter entry into the ureter.
6. *Catheter size:* Does not appear to be a predisposing risk factor, as misplacement reported with the smallest size of 12 Fr. and the largest size of 30 Fr. However, it’s important to note that this observation requires further clarification, as a significant portion of the data was missing.
7. Age, laterality, ADLs: Not relevant (further clarification is needed).

## 6. Key takeaway

What can be done if the balloon in the ureter is non-deflatable?^[48]^

➢ Deflate the balloon using conventional manual syringe aspiration.
➢ If these methods prove unsuccessful, alternatives such as cutting off the inflation channel, or bursting the balloon with a guidewire applied through the inflation channel.
➢ Seeking specialized medical assistance, consulting with an interventional radiologist or urologist may be necessary for ultrasound-guided/cysto-ureteroscopy balloon puncture.

How to prevent misplacement of a urinary catheter into the ureter?

➢ It is essential to be mindful of certain factors that increase the risk of misplacement, particularly in female patients, those with neurological disorders, neurogenic or contracted bladders, and individuals with long-term indwelling catheters.
➢ To confirm proper urinary catheter placement, it is advisable to ensure urine flow before inflating the balloon. Subjective resistance during inflation may indicate an inappropriate position.
➢ If the catheter is inserted without urine output, a saline flush is recommended to ensure the catheter is unobstructed.^[49]^
➢ For urethral catheters, verifying the appropriate length outside the urethra is crucial.
➢ Similarly, for suprapubic catheters, measuring the length of the initial catheter and marking the corresponding length on the replacement catheter helps ensure correct placement at the proper depth.^[50]^
➢ During balloon inflation, careful observation for any accompanying pain is necessary, although this may not be apparent in patients with sensory disorders.
➢ Consider using short-tip^[27,31]^ or blunt-tip urinary catheters, such as silicone nephrostomy catheters with an open-end, blunt-tip, and a smaller size balloon, as replacements for urethral catheters.
➢ At the conclusion of the procedure, gently withdrawing the catheter to the bladder neck is recommended for a safe process.^[21]^

What if inadvertent ureteral cannulation occurs?

1. If there is suspicion of catheter misplacement into the ureter, conducting an ultrasound examination as the initial diagnostic choice to determine its location and the presence of hydronephrosis. A contrast-enhanced CT scan, a noninvasive study, serves as a definitive tool to assess complications, particularly ureteral rupture. Exercise caution in cases involving patients with AKI.
2. Patients without ureteric injury may undergo conservative management, involving catheter change or removal, along with antibiotic treatment for UTIs.
3. In instances of ureteric rupture, urinary diversion becomes imperative, achieved through either the insertion of a ureteric stent or percutaneous nephrostomy. Surgical repair is warranted for severe ureteric injury.

## 7. Limitations

The limitation of this article is the small number of cases, with each article only able to present as a case report or case series. However, our review is the most comprehensive literature review to date.

## 8. Conclusion

This article presents a noteworthy case, coupled with an extensive literature review, shedding light on the complexities of ureteral misplacement. Early detection, intervention, and a multidisciplinary approach are crucial in cases of ureteral misplacement. It emphasizes the importance of considering patient risk factors, diagnostic approaches and employing appropriate techniques during catheterization procedures.

## Author contributions

**Investigation:** Jiun-Jia Li, Chin-Fong Au.

**Writing—original draft:** Jiun-Jia Li.

**Conceptualization:** Chin-Fong Au.

**Formal analysis:** Chin-Fong Au.

**Writing—review & editing:** Chin-Fong Au.
